# Microbial keratitis: a practical guide

**Published:** 2025-01-31

**Authors:** Astrid Leck

**Affiliations:** 1 Assistant Professor: International Centre for Eye Health, LSHTM, London, UK.

**Prompt diagnosis and treatment are critical for achieving a good outcome for patients.**

**Figure:**
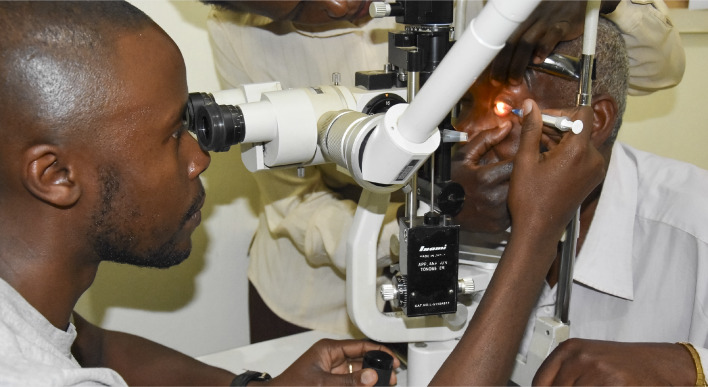
Taking a corneal scrape at the slit lamp. UGANDA

© GILBERT ARINDA CC BY-NC-SA 4.0

Microbial keratitis is a sight-threatening ocular emergency which continues to affect several million people worldwide each year, resulting in loss of vision and negatively impacting people's quality of life. In some countries, up to 10% of patients will lose the infected eye; as a result, they may experience depression, anxiety, social stigma, and increased poverty due to reduced employment, as highlighted in the case study overleaf.

Leading risk factors vary geographically and include ocular trauma, occupational exposure, contact lens wear, and pre-existing ocular surface disease. Corneal infections are challenging to manage, and good outcomes depend on prompt recognition, referral to a specialist eye unit for accurate diagnosis, and appropriate, intensive treatment.

The range of microorganisms responsible for microbial keratitis is diverse. Treatment is pathogen specific, so it is important to find out which microorganism is responsible and to test for susceptibility to different antimicrobial medications, to inform prescribing. However, the facilities, equipment, and personnel needed for diagnostic and susceptibility testing are often not available in places where the magnitude of disease is greatest. Many settings also lack evidence-based national treatment guidelines.

In this issue, we provide diagnostic and treatment guidance for all eye health workers, including specific recommendations for those working in settings where diagnostic laboratory support is either limited or not available. We also offer resources in the form of posters which can be adapted and reused to raise awareness among communities in settings where agriculture-related corneal abrasions are prevalent, and to provide advice for pharmacists, who are often a patient's first point of contact.

**CASE STUDY Tanzania**

Lemarti*, a Masaai student planning to study medicine at university, was diagnosed with fungal keratitis shortly before starting his final year of school. Although the keratitis eventually resolved, the resulting corneal scar affected his physical appearance and left him blind in one eye (visual acuity < 3/60).

As a result, he faced significant stigma: “Even the group that I used to discuss with began to neglect me, so I had to start studying alone.” This, together with the loss of income from farming – due to his need to wear spectacles – forced him to drop out of school. As Lemarti explained: “After I faced this problem and now that I use glasses, it means I can't go for grazing and dig. So that made me stop school because I couldn't raise enough money.”

Lemarti expressed a profound sense of uncertainty about his future. “I am concerned for my present and future too. If I fail to manage my present, what about my future?” The vision loss also affected Lemarti's standing in the community. “Everyone is looking at me… I am supposed to look for money, and get married, I am a young man,” he explained, adding that the condition left him unable to fulfill these responsibilities.

In addition to this feeling of exclusion, Lemarti described feeling uncomfortable in public places and lacking in confidence, explaining how this emotional burden was as debilitating as the physical effects of the condition.

Lemarti's case is just one of many patient stories that highlight the limitations of current medical counseling and treatment in settings where the magnitude of vision impairment is greatest. Ongoing support for patients who lose vision in one eye is critical for their welfare. Understanding the impact on patient's lives is also important in order to advocate for improved treatment options, including increased availability of appropriate and effective antimicrobial eye drops and corneal transplant services, which may provide better outcomes and hope for patients like Lemarti.

– **Zoha Mian**, MD Candidate: University of Louisville, USA

*
*Name has been changed*

